# Selective Enrichment of Two Different Types of *Nitrospira*-like Nitrite-oxidizing Bacteria from a Wastewater Treatment Plant

**DOI:** 10.1264/jsme2.ME12209

**Published:** 2013-05-09

**Authors:** Hirotsugu Fujitani, Yoshiteru Aoi, Satoshi Tsuneda

**Affiliations:** 1 Department of Life Science and Medical Bioscience, Waseda University, 2–2 Wakamatsu-cho, Shinjuku-ku, Tokyo, 162–8480, Japan; 2 Institute for Sustainable Science and Development, Hiroshima University, 2–313 Kagamiyama, VBL403, Higashi-Hiroshima, Hiroshima, 739–8527, Japan; 3 Department of Biology, Northeastern University, Mugar Lifescience Bldg. 313, Boston, MA 02115, USA

**Keywords:** continuous feeding bioreactor, nitrification, *Nitrospira*, uncultured bacteria

## Abstract

Nitrification is an important step in nitrogen removal in biological wastewater treatment processes. Recently, *Nitrospira* have been recognized as the numerically dominant nitrite-oxidizing bacterial genus primarily responsible for the second step of aerobic nitrification; however, *Nitrospira* usually resist cultivation under laboratory conditions and only one species enriched from activated sludge has been described. In this study, a novel enrichment method for *Nitrospira* was successfully developed using continuous feeding bioreactors. By controlling nitrite concentrations strictly in the bioreactor at low levels below 10 mg-N L^−1^, coexisting members of sublineages I and II of the genus *Nitrospira* were enriched selectively. The maximum ratios of sublineages I and II to total microbial cells achieved 88.3% and 53.8%, respectively. This enrichment method is potentially applicable to other uncultured *Nitrospira*.

Nitrite oxidation is an important step in nitrogen removal in biological wastewater treatment processes. The nitrite-oxidizing bacteria (NOB) previously identified as dominant in wastewater treatment plants belong to either the genus *Nitrobacter* or the genus *Nitrospira*. Traditionally, *Nitrobacter* were regarded as the key nitrite oxidizers because they were more frequently cultured in the laboratory from wastewater treatment plant biomass. However, cultivation-independent molecular methods indicated that members of the genus *Nitrospira*, and not *Nitrobacter* species, were numerically predominant and underwent nitrite oxidation in laboratory-scale nitrifying bioreactors and full-scale industrial wastewater treatment plants (2, 4, 11, 25). Subsequently, comparative analysis of 16S rRNA gene sequences revealed that the genus *Nitrospira* was classified into four different phylogenetic lineages and that sublineages I and II play a key role as nitrite oxidizers in wastewater treatment plants (5).

Although the physiological properties of the genus *Nitrospira* in nitrifying activated sludge or biofilms were investigated by molecular-based approaches (5, 19, 22, 26), detailed biochemical and genomic analyses of *Nitrospira* would require high enrichments or pure cultures; however, *Nitrospira* are usually recalcitrant to cultivation under laboratory conditions. Indeed, only five species within the genus *Nitrospira* have been described (7, 13, 14, 23, 27) and recently the whole genome sequencing analyses of one strain, which was named “*Candidatus* Nitrospira defluvii”, allowed insight into the key metabolic pathways of *Nitrospira* and its evolution (15). *Nitrospira* enrichment or pure cultivation requires several factors to be considered. First, it is well-known that *Nitrospira* are sensitive to the concentration of their substrate, nitrite. Indeed the growth activity of *Nitrospira* is inhibited at high nitrite concentrations (over 80 mg-N L^−1^) (18, 26). Second, out-competing *Nitrobacter* is essential for selective enrichment of *Nitrospira*. In a recent successful attempt, Spieck *et al.* (23) repressed the growth of *Nitrobacter* cells and heterotrophic contaminants by using low nitrite and adding antibiotics (ampicillin) to the growth medium, resulting in the selective enrichment of *Nitrospira* sublineage I. Meanwhile, it was reported that nitrite concentration influenced the population of *Nitrospira* sublineages I and II, and that ecological differentiation occurred within the genus *Nitrospira* (16). However, the members of sublineage II, which have been recognized as the other major players of the genus *Nitrospira* in wastewater treatment plants, have not yet been enriched and are incompletely characterized, except for *Nitrospira moscoviensis*, isolated from a Moscow heating system (7).

Here, we hypothesized that controlling nitrite concentration could manipulate the population of coexisting members of sublineages I and II of the genus *Nitrospira* and enrich either members selectively. The objective of this study was selective enrichment of uncultured nitrite-oxidizing bacteria of the genus *Nitrospira* using a continuous feeding bioreactor. The design of the bioreactor and the operational condition aimed to achieve selective enrichment; namely 1) supplying the inlet medium solution at a high flow rate to effectively discharge metabolic by-products and minimize the growth of heterotrophs; 2) maintaining low nitrite concentrations in the bioreactor, enabling *Nitrospira* to out-compete *Nitrobacter*; 3) maintaining nitrite concentrations strictly at lower levels in the bioreactor to control the population within the genus *Nitrospira*.

## Materials and Methods

### Sampling and Primary enrichment

**Fig. 1:**
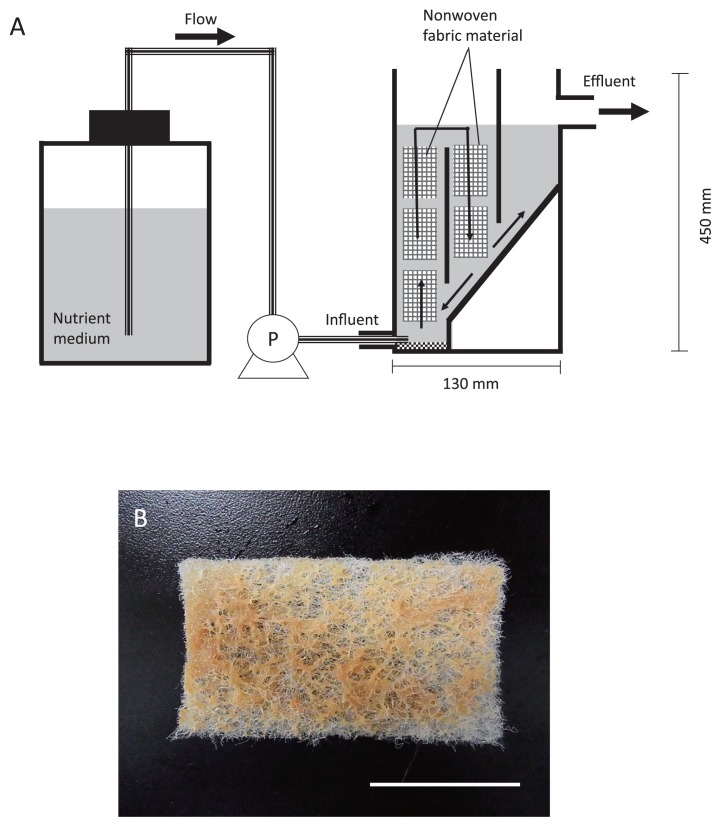
(A) Schematic drawing of a continuous feeding bioreactor for selective enrichment of the genus *Nitrospira*. (B) Biomass attached to biomass carriers comprising porous polyester nonwoven fabric materials. Scale bar is 4 cm.

The primary inoculum for the enrichment of *Nitrospira* was sampled from the nitrification stage of the municipal wastewater treatment plant in Ochiai, Tokyo, Japan, in February 2009. The samples were transported to the laboratory in an opaque container to prevent exposure to light. Upon receipt of the samples, a continuous feeding bioreactor with biomass carriers comprising porous polyester nonwoven fabric materials (0.7 cm thickness; Japan Vilene, Tokyo, Japan) was set up to retain the bacteria in an active state (Fig. 1). The volume of the bioreactor was 2 L. The temperature was maintained at 23°C and the pH was adjusted to between 7.8 and 8.3 inclusive. The culture was continuously aerated to provide excess oxygen. The inorganic medium comprised NaNO_2_ (24.6–394 mg L^−1^), K_2_HPO_4_ (38.2 mg L^−1^), MgSO_4_·7H_2_O (61.1 mg L^−1^), CaCl_2_·2H_2_O (10 mg L^−1^), FeSO_4_·7H_2_O (5 mg L^−1^), MnSO_4_·5H_2_O (54.2 μg L^−1^), H_3_BO_3_ (49.4 μg L^−1^), ZnSO_4_·7H_2_O (43.1 μg L^−1^), Na_2_Mo_4_O_4_ (27.6 μg L^−1^), and CuSO_4_·5H_2_O (25 μg L^−1^). The influent nitrite-nitrogen concentration was periodically increased from 5 to 80 mg L^−1^ for two years. Approximately half of the biomass was manually discharged from the bioreactor every 0.5–1 month in order to reduce over-grown biomass such as inactive cells, debris, and extracellular polymeric substances. During the same time periods, the hydraulic retention time (HRT) was adjusted to between 1.6 and 4.8 h. Subsequently, a portion of enrichment culture was transferred to two bioreactors, Reactors I and II, in order to enrich sublineages I and II of the genus *Nitrospira* selectively.

### Selective enrichment of sublineages I and II

Selective enrichment within the genus *Nitrospira* was performed by controlling nitrite concentration in the bioreactors. Detailed experimental conditions of the two bioreactors are described in Table 1. In Reactor I, the influent nitrite concentration was 50 mg-N L^−1^ and nitrite concentration in the bioreactor was maintained below 10 mg-N L^−1^ (Phase A). After day 237, the influent nitrite concentration was controlled between 10 and 40 mg-N L^−1^ in order to keep nitrite concentration in the bioreactor below 1 mg-N L^−1^ (Phase B). In Reactor II, the influent nitrite concentration was constantly 40 mg-N L^−1^, with the result that nitrite concentration in the bioreactor was below 2 mg-N L^−1^ (Phase C) or 1 mg-N L^−1^ (Phase D). After day 334, the nitrite concentration in the influent and in the bioreactor was maintained in the same manner as in Phase A (Phase E). The media used in both bioreactors were the same as described in primary enrichments except for nitrite concentration. The temperature was maintained at 23°C and the pH was adjusted to between 7.8 and 8.3. The culture was also continuously aerated to provide excess oxygen.

### Chemical Analyses

Nitrite-nitrogen and nitrate-nitrogen concentrations were determined quantitatively by ion chromatography (IC 2001; Tosoh, Tokyo, Japan). Influent/effluent samples in the bioreactor and nitrite oxidation activity test samples were filtered through 0.20 μm cellulose acetate membrane filters (Advantec, Tokyo, Japan).

### Fluorescence in situ hybridization (FISH) and DNA staining

All *in situ* hybridizations were performed following the standard protocol (1) in hybridization buffer at 46°C for 2.5 h. The applied oligonucleotide probes were S-G-Ntspa-0662-a-A-18 (specific for the genus *Nitrospira*; 5), S-*-Ntspa-1431-a-A-18 and S-*-Ntspa-1151-a-A-20 (specific for sublineages I and II of *Nitrospira*, respectively; 16), and S-G-Nbac-1035-a-A-18 (NIT3, specific for the genus *Nitrobacter*; 25). Oligonucleotides were synthesized and fluorescently labeled with a hydrophilic sulfoindocyanine dye (Cy3) or fluorescein isothiocyanate (FITC) at the 5′ end (Tsukuba Oligo Service, Tsukuba, Japan). The *Nitrospira*- and *Nitrobacter*-specific probes and competitor oligonucleotides were used in separate hybridization experiments as described by Daims *et al.* (5) and Wagner *et al.* (25). SYTOX Green nucleic acid stain (Life Technologies, Carlsbad, CA, USA) was applied as a universal cellular stain. Stained cells were detected and recorded using a confocal laser scanning microscope (IX71; Olympus, Tokyo, Japan) and by a fluorescence microscope (Axioskop 2 plus; Carl Zeiss, Oberkochen, Germany).

The ratio of *Nitrospira* to the total microorganisms in enrichment samples was quantitatively analyzed with a microscopic direct counting method. Most of the biomass attached to nonwoven fabric materials in the bioreactor could be retrieved manually and comparatively easily. To estimate the relative abundance of *Nitrospira* in the bioreactor correctly, biomass was retrieved from all nonwoven fabric materials in the bioreactor. The average cell numbers were determined from at least 10 representative microscopic images of enrichment samples with triplicate measurements.

### DNA extraction and polymerase chain reaction (PCR) amplification

DNA was extracted from primary enrichment samples using the ISOIL extraction kit (Nippon Gene, Tokyo, Japan) according to the manufacturer’s instructions. The 16S rRNA gene fragment (*ca.* 1,500 bp) of the total DNA was amplified using the primers 27f/1492r. The following thermal profiles were used in the 16S rRNA gene amplification: an initial denaturing step conducted at 95°C for 2 min, followed by 30 cycles of denaturation at 94°C for 1 min, annealing at 55°C for 1 min, and elongation at 72°C for 1 min. The final extension step was conducted at 72°C for 2 min. The PCR reaction mixtures (50 μL) contained 10×PCR buffer, 20 μM of each primer, 2.5 mM of dNTPs, and *Ex Taq* DNA polymerase (Takara Bio, Otsu, Japan).

### Cloning of the 16S rRNA gene and phylogenetic analysis

Amplified PCR products were purified using the Wizard SV Gel and PCR Clean up System (Promega, Tokyo, Japan). Purified PCR products were cloned into the pGEM-T easy vector system (Promega) and transformed into competent cells following the manufacturer’s instructions. To assess the quality and size of inserts, colony PCR using vector-specific M13 primers was carried out using EmeraldAmp PCR Master Mix (Takara). The cloned 16S rRNA gene fragments were sequenced using primers 27f/1492r from Fasmac. Finally, the 16S rRNA gene sequences, comprising approximately 1,500 bases, were determined. Alignment editing and phylogenetic analyses were performed using ChromasPro version 1.4.1 software (Technelysium Pty, Tewantin, Australia) and MEGA4.0 software (24). The bacterial 16S rRNA gene sequences were compared with those available from the DNA Data Bank of Japan (DDBJ) database. All clones containing 16S rRNA genes with >98% similarity to the above-characterized sequence were grouped into an operational taxonomic unit. Genetic distance was calculated using a *p*-distance model of nucleic acid substitution.

### Growth activity test

To determine the specific growth rate of sublineages I and II of the genus *Nitrospira*, a part of the enrichment culture in Reactor II on the 260th day was incubated in batch cultures for one month. The media used in the batch cultures were the same as described in the continuous feeding bioreactors except for nitrite concentration. All incubations were performed in 300 mL Erlenmeyer flasks containing 100 mL fresh nitrite medium (2 or 10 mg-N L^−1^) at 23°C. The volume of 1 mL fresh nitrite medium was added to prevent the depletion of nitrite in case nitrite concentration was below one-tenth of the set values (0.2 or 1 mg-N L^−1^). The number of *Nitrospira* cells during incubation was determined by microscopic direct counting after FISH analysis.

## Results

### Primary enrichment

**Fig. 2:**
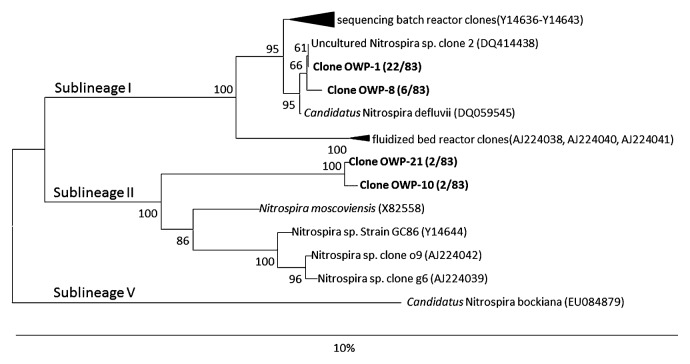
Phylogenetic analysis showing the affiliation of clones from primary enrichment. The phylogenetic tree is based on 16S rRNA gene sequences of selected *Nitrospira*. The tree was constructed using the neighbor-joining algorithm. Numbers at the branch nodes are bootstrap values. Previously defined sublineages (5) of the genus *Nitrospira* (Roman numbers) are shown. Scale bar corresponds to 10% estimated sequence divergence.

In this study, enriched cultures of *Nitrospira* were prepared from February 2009 for subsequent selective enrichment of sublineages I and II. As a result, *Nitrospira* were enriched efficiently in a continuous feeding bioreactor (constituting 96% of the total microbial cells at maximum) and the populations were retained stably (exceeding 80% of the total microbial cells) for over 2 years. The 16S rRNA gene clone library constructed from primary enrichment revealed that 32 clones (from 83 clones) were related to *Nitrospira* (28 clones of sublineage I and 4 clones of sublineage II) (Fig. 2), while the other clones were related closely to heterotrophic microorganisms within the phyla *Proteobacteria*, *Bacteroidetes*, *Acidobacteria*. Clones of sublineage I from enrichment samples in this study (OWP-1 and OWP-8) showed a high sequence similarity (99.6–99.8%) to *Candidatus* Nitrospira defluvii (23). OWP-10 and OWP-21 clones were distantly related to *Nitrospira moscoviensis*, the only described species in sublineage II with 95.6–95.8% similarity (7). On the basis of FISH analysis, the ratios of *Nitrospira* sublineage I and sublineage II to the total microbial cells in the primary enrichment (at day 0 of subsequent experiment) were 75.2% and 11.0%, respectively.

### Selective enrichment of sublineages I and II

Two fixed-bed continuous feeding bioreactors were designed to selectively enrich sublineages I and II of the genus *Nitrospira* (Fig. 1A). A portion of the primary enrichment was transferred to the two bioreactors to investigate the population structure of *Nitrospira*. The bioreactor carriers comprised nonwoven fabric materials for the retention of *Nitrospira* cells (Fig. 1B), while synthetic medium was fed to the bioreactors at a high flow rate. Approximately half of the biomass was manually discharged from the bioreactors every 0.5–1 month in order to reduce over-grown biomass such as inactive cells, debris, and extracellular polymeric substances, thereby increasing the ratio of *Nitrospira* cells to other microorganisms. The number of *Nitrospira* and other microbial cells was monitored by FISH direct counting throughout the experiment.

The nitrite concentration in the two bioreactors was controlled in order to manipulate populations of two types of *Nitrospira*, sublineages I and II, as shown in Table 1. Previous studies (6, 16) found that nitrite concentration influenced the population of *Nitrospira* sublineages I and II. On the basis of data from previous studies, we hypothesized that controlling nitrite concentration strictly in the bioreactors could manipulate the population of coexisting members of sublineages I and II of the genus *Nitrospira* and enrich other members selectively.

The manipulation of nitrite concentration in the bioreactor was performed by controlling the flow rate of the inlet medium or nitrite concentration in the inlet medium. In Reactor I, a relatively high concentration of nitrite (2–6 mg-N L^−1^) was maintained initially (Phase A) in order to selectively enrich and maintain *Nitrsopira* sublineage I. Phase A was followed by Phase B, shifting to relatively low nitrite concentration (0–1 mg-N L^−1^) to induce a population change from sublineage I to II. In Reactor II, an intermediate level concentration of nitrite (0–2 mg-N L^−1^) was maintained initially (Phase C) to try and balance populations of sublineages I and II. Phase C was followed by Phase D, in which the nitrite concentration was kept at a relatively low level (0–1 mg-N L^−1^) to increase the ratio of sublineage II. Finally, shifting to a relatively high concentration of nitrite in the bioreactor demonstrated the recovery of the ratio of sublineage I during Phase E. The transition of the two populations in each reactor is described below.

**Fig. 3:**
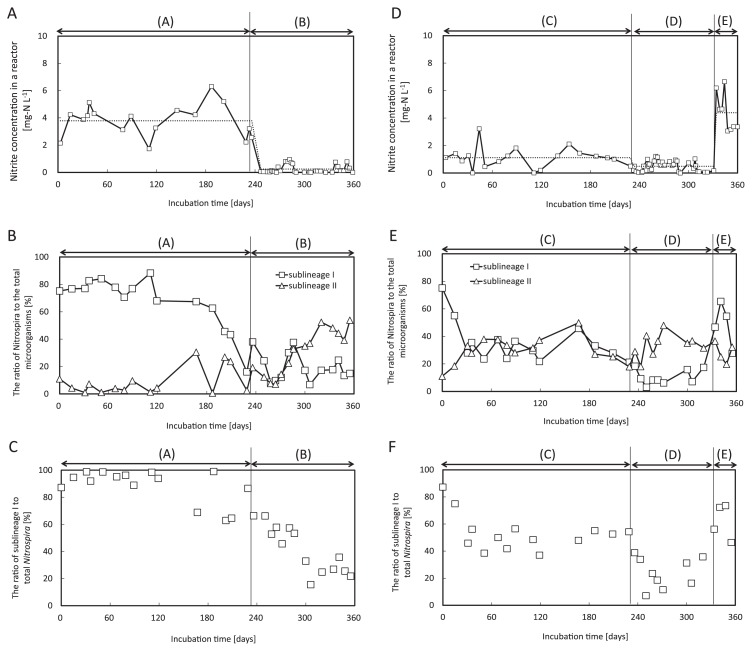
Competition between sublineages I and II of the genus *Nitrospira* in two continuous feeding bioreactors (A–C: Reactor I; D–F: Reactor II). (A) and (D) Open square represents nitrite-nitrogen concentration in the bioreactor; dotted line represents average nitrite-nitrogen concentration in the bioreactor at each phase. (B) and (E) Ratio of *Nitrospira* to total microorganisms, calculated by FISH analysis with direct counting. (C) and (F) Ratio of sublineage I to total *Nitrospira*.

**Fig. 4:**
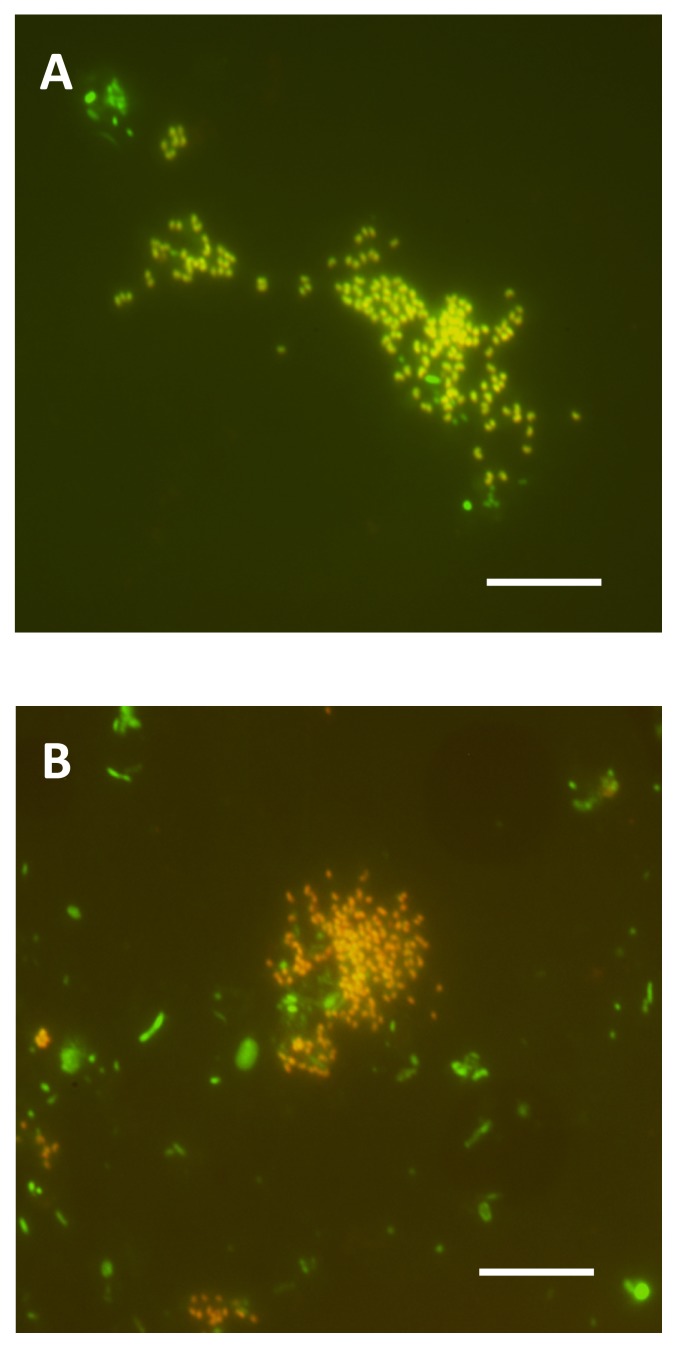
Fluorescence *in situ* hybridization (FISH) images of enrichment culture. (A) Enrichment sample of sublineage I in Reactor I on 111 day. (B) Enrichment sample of sublineage II in Reactor I on 355 day. *In situ* hybridization was performed with Cy3-labeled probe Ntspa1431, specific for the detection of *Nitrospira* sublineage I (red) and Cy3-labeled probe Ntspa1151, specific for the detection of *Nitrospira* sublineage II (red). Green cells were stained only with SYTOX green, which stains all the cells. Yellow signals resulted from binding both the Cy3-labeled probe and SYTOX green to one cell. Both scale bars are 5 μm.

In Reactor I, the average nitrite concentration was kept constant at about 3.8 mg-N L^−1^ until day 236 (Phase A in Fig. 3A). The population of *Nitrospira* sublineage I was maintained at high levels for the first six months followed by a decrease along with the decrease of nitrite concentration in the bioreactor (Phase A in Fig. 3B and C). From 236 days, the nitrite concentration in the bioreactor was controlled below 1 mg-N L^−1^ (0.24 mg-N L^−1^ on average) (Phase B in Fig. 3A). During this period, a gradual shift in the population of *Nitrospira* was observed. The ratio of *Nitrospira* sublineage II to total *Nitrospira* cells gradually increased and reached around 80% after 300 days (*vice versa* for sublineage I) (Fig. 3C). The maximum ratio of sublineages I and II to total microbial cells was 88.3% on day 111 and 53.8% on day 355, respectively (Fig. 3B). FISH images at this maximum ratio are shown in Fig. 4. *Nitrobacter*, considered a critical competitor of *Nitrospira* (3, 9, 18), were not detected at all by FISH throughout the experiment.

In Reactor II, nitrite concentration was controlled below 2 mg-N L^−1^ (1.1 mg-N L^−1^ on average) until day 231 (Phase C in Fig. 3D). In the first month, the ratio of *Nitrospira* sublineage I markedly decreased and reached around 50% of total *Nitrospira* cells (*vice versa* for sublineage II). After this transition, the population dynamics of sublineages I and II achieved stable equilibrium (Phase C in Fig. 3F). In Phase D, nitrite concentration in the bioreactor was controlled below 1 mg-N L^−1^ (0.49 mg-N L^−1^ on average). As a result, the population of sublineage II became more dominant than that of sublineage I in response to the change of the operation condition in the bioreactor. After day 334, an abrupt increase of nitrite concentration in the bioreactor resulted in the opposite population in Phase D (Phase E in Fig. 3D and F).

### Relationship between nitrite concentration and population structure of *Nitrospira*

**Fig. 5:**
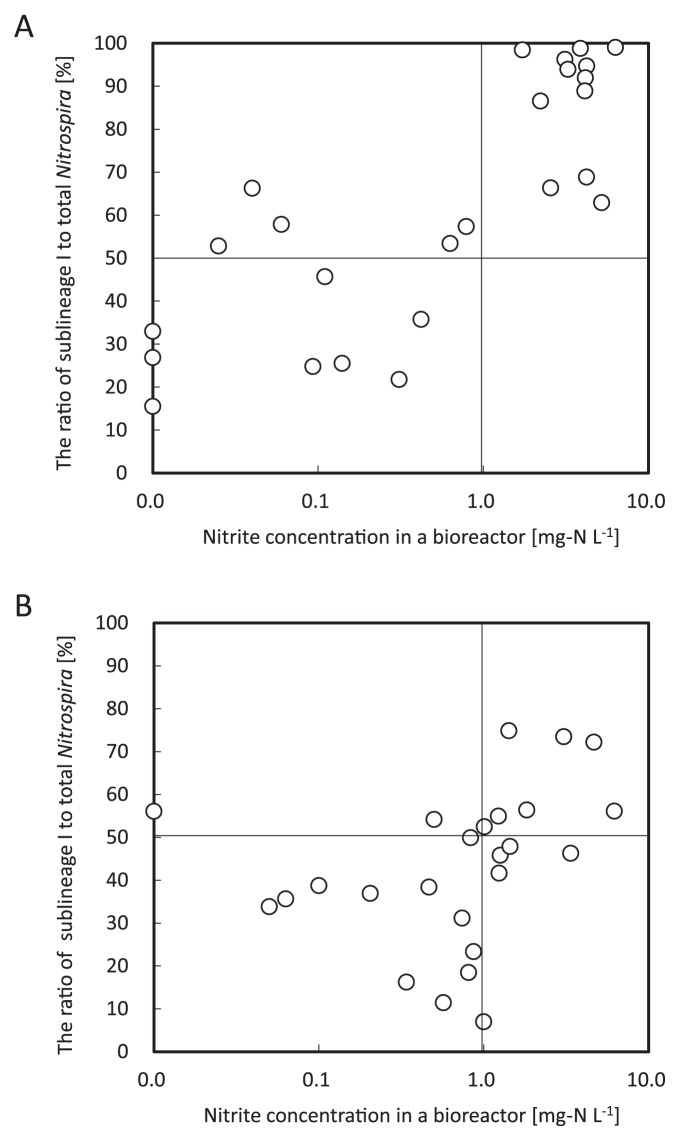
Relationship between nitrite concentration in a bioreactor and the ratio of sublineage I to the total *Nitrospira*. (A) Each dot indicates the values measured in Reactor I on the following days: 15, 31, 36, 79, 89, 111, 119, 167, 187, 202, 229, 236, 250, 258, 264, 271, 280, 286, 300, 306, 320, 334, 341, 348, 355. (B) Each dot indicates the values measured in Reactor II on the following days: 15, 31 36, 51, 68, 79, 89, 119, 167, 187, 209, 229, 236, 243, 250, 258, 264, 271, 300, 306, 320, 334, 341, 348, 355. If the observed values were below detection limits in the measurement of ion chromatography, nitrite concentration was assumed to be 0 mg-N L^−1^.

To assess the relationship between the ratio of sublineage I or II and nitrite concentration in a bioreactor, a correlation diagram was drawn from items investigated in the bioreactor (Fig. 5). When correlating the nitrite concentration and population structure of *Nitrospira*, the incubation time required to change the population in response to nitrite concentration was estimated to be within 1–2 days under the continuous feeding system in this study. In Reactor I, different tendencies were clearly observed with the boundary of 1 mg-N L^−1^ nitrite concentration in the bioreactor (Fig. 5A). With high nitrite concentration over 1 mg-N L^−1^, the population of sublineage I dominated absolutely whereas with lower nitrite concentration below 1 mg-N L^−1^, its dominance collapsed. In Reactor II, it was clearly indicated that the population of sublineage I decreased with lower nitrite concentration below 1 mg-N L^−1^ (Fig. 5B).

### Growth activity test

**Fig. 6:**
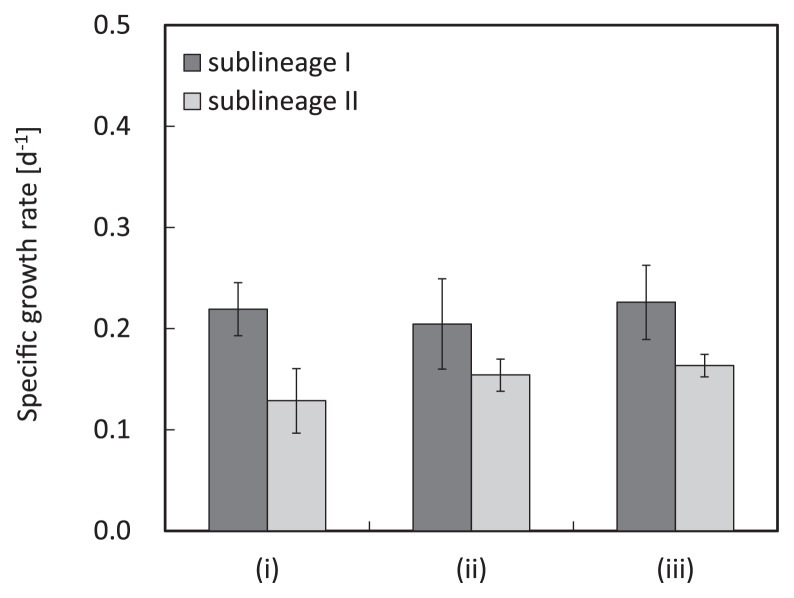
Growth rates estimated from time-dependent changes in the *Nitrospira* populations in batch cultures for one month of incubation. Cell numbers were estimated by FISH analyses and microscopic direct counts. Experimental conditions were as follows: (i) nitrite concentration was 2 mg-N L^−1^, static culture; (ii) nitrite concentration was 10 mg-N L^−1^, static culture; (iii) nitrite concentration was 10 mg-N L^−1^, shaking culture. Error bars indicate the standard deviation of the mean of triplicate measurements.

To determine the specific growth rate of sublineages I and II of the genus *Nitrospira*, a part of the enrichment culture in the Reactor II on day 260 was incubated in batch cultures for one month. The cell numbers of *Nitrospira* sublineages I and II at culture start-up were 1.49×10^5^ and 9.36×10^5^ cells mL^−1^, respectively. After 15 days of incubation, both cell numbers increased up to 10^6^–10^7^ order level under all experimental conditions. Subsequently, the specific growth rates of sublineages I and II were estimated from the time-dependent changes in the *Nitrospira* populations in the batch cultures. The specific growth rate of sublineage I was higher than that of sublineage II under any experimental conditions. The average growth rates of sublineages I and II were 0.22 and 0.15 d^−1^, respectively (Fig. 6), which were lower than the values of the maximum specific growth rate estimated by mathematical modeling in the previous study (sublineages I and II were 2 and 0.75 d^−1^, respectively; 16).

## Discussion

This work describes the selective enrichment of uncultured nitrite-oxidizing bacteria of the genus *Nitrospira*, which are of major importance for nitrogen removal in wastewater treatment plants. We have developed a novel method for enrichment culture using continuous feeding bioreactors and enriched selectively sublineages I and II within the genus *Nitrospira* by regulating strictly nitrite concentration in the bioreactor. To achieve the aim as described above, the following three factors must be considered: 1) minimizing the growth of heterotrophs, 2) suppressing *Nitrobacter* as competitors, 3) controlling the population of sublineages I and II.

It is generally known that a considerable number of various types of heterotrophic bacteria coexist with nitrifying bacteria despite providing an inorganic substrate that does not contain any organic matter. Organic metabolic byproducts are released from active nitrifying bacteria and cell lysate is generated from dead cells, which provide carbon and energy sources for heterotrophs (12, 17, 20, 21); therefore, minimizing heterotrophic growth is an important issue for the enrichment of *Nitrospira*. Indeed, supplying the inlet medium solution at a high flow rate and discharging overgrown biomass periodically were effective to reduce the quantity of inactive cells, debris, and extracellular polymeric substances in the biomass that constitutes a potential growth substrate for heterotrophs. Moreover, the nonwoven fabric materials used in this study were effective to capture and retain biomass stably in the bioreactor even under high flow rate conditions. This is especially effective to enrich slow-growing microorganisms, as proved in previous studies that demonstrated the enrichment of anaerobic ammonium-oxidizing (anammox) bacteria (8, 10).

To acquire a highly enriched culture of *Nitrospira*, it is necessary to enhance the growth activity of *Nitrospira* maximally by inhibiting its competitors, *Nitrobacter*. Early studies demonstrated that the affinity for nitrite is higher in *Nitrospira* than in *Nitrobater*; thus, *Nitrospira* are considered as k-strategists while *Nitrobacter* are r-strategists (assuming that nitrite concentration is the major driver of the competition; 3, 22, 26). Additionally, it is well-known that *Nitrospira* are sensitive to nitrite concentration. The growth activity of *Nitrospira* is inhibited at high nitrite concentrations (over 80 mg-N L^−1^) (18, 26). Indeed, feeding low levels of nitrite into the reactor throughout the enrichment process proved effective at out-competing *Nitrobacter* in this study.

Manipulating the population ratio between sublineages I and II within the genus *Nitrospira* was another challenge. This was accomplished by strictly regulating nitrite concentration in the bioreactor within the range of 0–10 mg-N L^−1^. Previous studies suggested the hypothesis that *Nitrospira* in sublineage II have higher affinity for nitrite than these organisms in sublineage I (6, 16). Actually, the population of the genus *Nitrospira* was changed markedly by controlling nitrite concentration in the bioreactor. The ratio of *Nitrospira* sublineage I was maintained at high levels with high nitrite concentration in the bioreactor (2–6 mg-N L^−1^) whereas the ratio of *Nitrospira* sublineage II increased under low nitrite concentration (0–1 mg-N L^−1^) (Fig. 5). Moreover, growth activity tests demonstrated that the specific growth rate of sublineage I was higher than that of sublineage II in batch cultures containing inorganic nitrite media (2 or 10 mg-N L^−1^) (Fig. 6); therefore, the above-described hypothesis was followed up in this study. Interestingly, the coexisting condition of sublineages I and II was found and maintained sustainably over 200 days with medium nitrite concentration (1.1 mg-N L^−1^ on average) (Phase C in Fig. 3F). It is probable that this coexisting population of sublineages I and II could occur only with the range of fairly limited nitrite concentration.

Meanwhile, the maximum enrichment level gave different results for sublineages I and II. The population of *Nitrospira* sublineage I to total microbial cells reached as high as 88.3% in Reactor I on day 111 (Phase A in Fig. 3B), whereas the ratio of *Nitrospira* sublineage II reached only 53.8% in Reactor I on day 355 (Phase B in Fig. 3B); therefore, highly enriched cultures of sublineage II could not be obtained in comparison with sublineage I. This result might be attributed to differences in the growth rate of sublineages I and II (Fig. 6). Since no external organic carbon was added to the bioreactor, the metabolic by-products produced by *Nitrospira* are the sole organic substrates supporting heterotrophic bacterial growth; therefore, the growth of heterotrophic bacteria was dependent on *Nitrospira*. Assuming that the amount of organic matter produced per bacterial cell division in sublineage II was higher than that of sublineage I, while the growth rate of sublineage II was lower than sublineage I in the present study, the relative abundance of *Nitrospira* to total microorganisms differed between sublineages; that is, the lower growth rate and higher production of organic matter synergistically lowered the relative abundance of sublineage II to total microorganisms compared with sub-lineage I-dominant biomass.

Challenging attempts to isolate members of *Nitrospira* sublineages I and II from enrichment samples obtained in this study are currently in progress. However, there are some difficulties in purification because *Nitrospira*-like bacteria in enrichment samples from activated sludge have a tendency to form multi-species aggregates with other microorganisms. Although selective enrichment of *Nitrospira* sublineage I was achieved successfully by using low nitrite and adding antibiotics (ampicillin) to the growth medium (23), there was still no pure culture in this group; therefore, alternative approaches to purification might be required.

In summary, we have enriched sublineages I and II selectively within the genus *Nitrospira* using continuous feeding bioreactors. Controlling nitrite concentration in the bioreactor at a low level was effective to culture these fastidious and slow-growing microorganisms. Development of a selective enrichment method for uncultured bacteria is significantly important for the isolation of as yet uncultured species, which will provide further physiological, biochemical, and genomic data.

## Figures and Tables

**Table 1:** Experimental conditions of the continuous feeding bioreactors

Reactor	Phase	Opearation Day	Average flow rate [L d^−1^]	Nitrite concentration [mg-N L^−1^]	

				Influence	Average in a bioreactor
I	A	0–236	13.1	50	3.8
	B	237–359	15.3	10–40	0.24

II	C	0–231	9.9	40	1.1
	D	232–333	9.5	40	0.49
	E	334–359	14.6	50	4.4
